# Transarterial coil embolization of an aortic root pseudoaneurym in a patient with Loeys-Dietz syndrome: a case report

**DOI:** 10.1186/s42155-020-00184-5

**Published:** 2020-12-08

**Authors:** Robert D’Ortenzio, Leandro Cardarelli-Leite, Ravjot Dhatt, Jacqueline Saw, Manraj Heran

**Affiliations:** 1 Department of Radiology, University of British Columbia, 11th Floor, 2775 Laurel St., Vancouver, V5Z 1M9 Canada; 2 Department of Radiology, Western University, 1151 Richmond St, London, N6A 3K7 Canada; 3 Department of Cardiology, University of British Columbia, 9th Floor, 2775 Laurel St., Vancouver, V5Z 1M9 Canada; 4 Department of Radiology, BC Children’s Hospital, 4500 Oak St., Vancouver, BC V6H 3N1 Canada

**Keywords:** Aortic aneurysm, Aortic pseudoaneurysm, Bentall, Interventional procedures, Loeys-Dietz, Connective tissue disease, Coil embolization, Cardiology

## Abstract

**Background:**

Loeys-Dietz syndrome (LDS) is a rare autosomal-dominant connective tissue disorder characterized by arterial aneurysms and vascular friability. Surgical intervention for LDS patients carries significant morbidity and mortality. Currently, the standard management of aortic root pseudoaneurysms is surgical intervention.

**Case presentation:**

A 20 year old male with LDS presented with a progressively enlarging ascending aortic aneurysm. He underwent a Bentall-type aortic root replacement complicated by a 20 mm aortic root anastomotic pseudoaneurysm. Due to the patient’s high risk for repeat surgical intervention, he underwent successful transarterial coil embolization of his aortic root pseudoaneurysm without complication.

**Conclusions:**

Coil embolization may provide an alternative treatment for patients presenting with aortic root pseudoaneurysm who are high risk for traditional surgical treatment, such as those with connective tissue disease.

## Background

Loeys-Dietz syndrome (LDS) is an autosomal-dominant connective tissue disorder first described in 2005. The most morbid sequalae of LDS are aortic aneurysm and dissection (Loeys et al. 2006).

The Bentall operation involves a mechanical aortic valve replacement with a composite aortic graft, and remains first line treatment for enlarging aortic root aneurysms (Bentall and De Bono 1968). However, there is a significant incidence of pseudoaneurysm following this surgery in the general population, ranging from 8 to 10% (Kouchoukos et al. 1991).

We describe a 20-year-old male with LDS who previously underwent a modified valve sparing Bentall type procedure for an expanding aortic root aneurysm who subsequently developed an enlarging pseudoaneurysm at the aortic root surgical anastomosis. We describe a percutaneous transarterial coil embolization technique for the management of a known complication of this rare disease.

## Case report

**Fig. 1:**
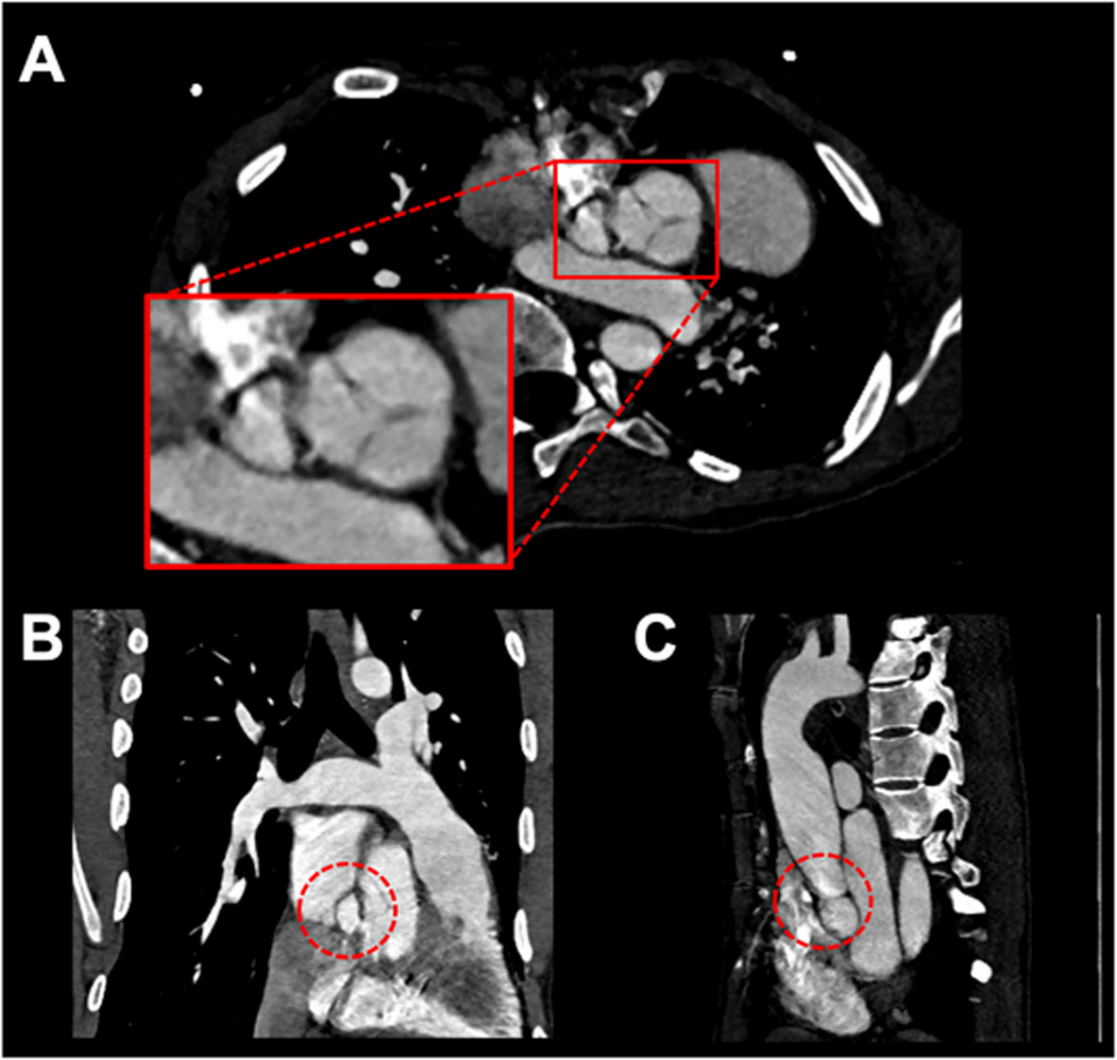
Cardiac Computed tomography (**a**: axial, **b**: coronal and **c**: sagittal reformats) demonstrates an enlarging 20 × 17 × 12 mm pseudoaneurysm arising from the aortic annulus surgical anastomosis caudal to the non-coronary cusp with neck measuring 3 mm post Bentall procedure (red circle)

A 20-year-old male with LDS initially presented with a progressively enlarging ascending aortic aneurysm measuring up to 4.2 cm at the sinotubular junction. Multidisciplinary team decision was made to proceed with a surgical trans-sternal aortic root Bentall-type replacement with a 28 mm diameter graft. No immediate complications were observed. The 1-month follow-up cardiac CT demonstrated a new aortic outflow tract pseudoaneurysm with the sac measuring 20 × 17 mm in axial and 12 mm in craniocaudal dimensions, with a narrow neck measuring 3 mm. The pseudoaneurysm originated from the proximal anastomosis of the graft below the non-coronary cusp of the aortic valve and extended between the left and right atria (Fig. 1). Echocardiography demonstrated dynamic caliber change of the pseudoaneurysm, distending during systole, and shrinking during diastole. An interdisciplinary discussion between cardiac surgery, cardiology, interventional radiology took place. After consultation with the patient’s family, it was felt that due to the high risk of repeat surgery, the decision was made to manage the pseudoaneurysm conservatively with short interval imaging follow up. However, a subsequent CTA 2 months following surgery demonstrated interval increase in size of the pseudoaneurysm (Fig. 1), thus, endovascular embolization was recommended.

**Fig. 2:**
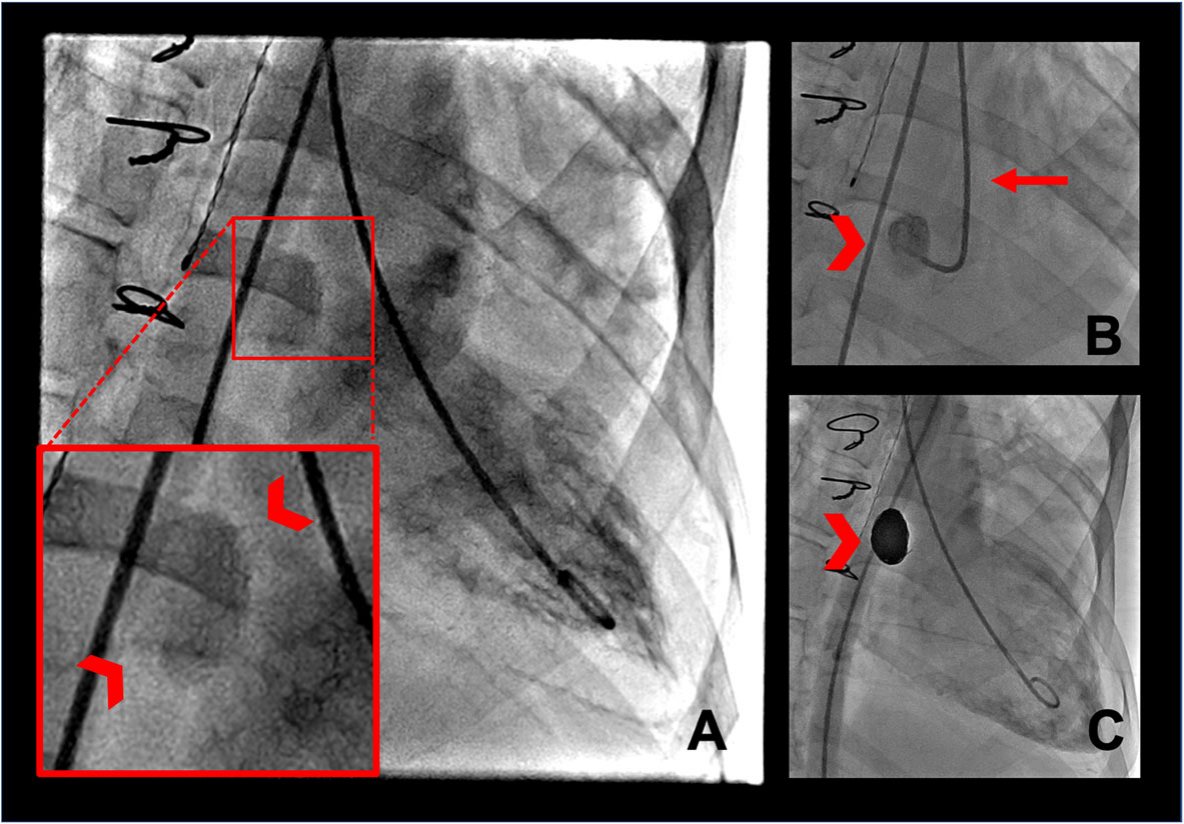
**a** Angiographic left ventriculogram remonstrates the aortic root pseudoaneurysm (red circle). **b** Successful catheterization of the pseudoaneurysm sac using the 6 French IMA catheter (red arrowhead). **c** Follow up left ventriculogram demonstrates no appreciable filling of the pseudoaneurysm post-coiling (red arrowhead)

The procedure was performed under general anesthesia under normotensive conditions. Interventional cardiology and interventional radiology performed the procedure in a joint fashion. Through a 6-French sheath inserted in the right common femoral artery, a pigtail catheter was advanced into the left ventricle. Left ventriculogram demonstrated the aortic pseudoaneurysm with its neck arising just inferior to the aortic valve (Fig. 2a). Engaging the pseudoaneurysm neck required several attempts due to its proximity to the aortic valve. Stable position was eventually achieved using an IMA 6-French catheter (Performa®; Merit Medical, Salt Lake, UT, USA) with advancement of an exchange length 300 cm coronary wire (High-Torque Balance Middleweight; Abbott, Abbott Park, IL, USA) into the pseudoaneurysm, followed by advancement of a microcatheter (Velocity®; Penumbra, Alameda, CA, USA) into the sac neck. A total of 12 detachable microcoils (PC 400 Coil®; Penumbra, Alameda, CA, USA) were used for embolization of the pseudoaneurysm, beginning with a 22 mm diameter × 60 cm long Complex Standard “framing” coil, and subsequent filling of the pseudoaneurysm using a combination of standard and soft complex shaped coils ranging down to 9 mm in diameter. Post-embolization left ventriculogram demonstrated no residual filling of the aneurysm (Fig. 2c). Hemostasis at the femoral arterial puncture site was then achieved using a closure device (Angio-Seal®, Terumo, Shibuya City, Tokyo, Japan).

**Fig. 3:**
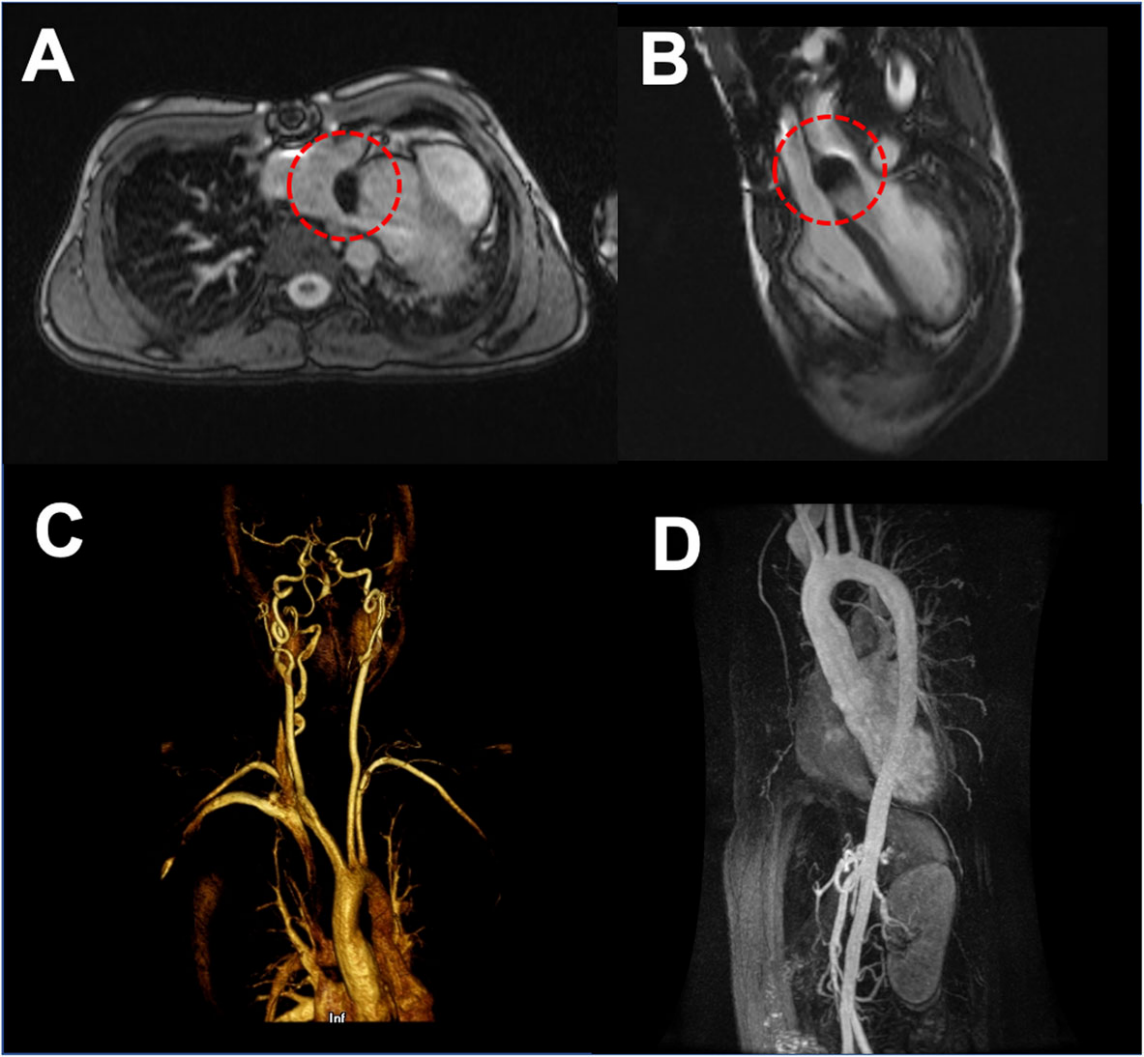
**a** Axial and **b** two chamber reformatted cardiac MR approximately 1 month post-procedure demonstrates no residual or recurrent pseudoaneurysm. Red circle: signal loss related to pseudoaneurysm coil embolization. **c** 3D and **d** time of flight MR angiography demonstrate no residual or recurrent pseudoaneurysm formation

The patient was extubated and admitted to the cardiac intensive care unit for observation. No immediate post operative complications were observed. The patient was discharged after 2 days of clinical monitoring. Follow-up cardiac MRI 1 month as well as 1 year postoperatively demonstrated no residual or recurrent aneurysm (Fig. 3). Repeat echocardiography 4 years later did not show recurrence of the pseudoaneurysm.

## Discussion

LDS is a rare, multi-system connective tissue disorder caused by an autosomal dominant, heterozygous mutation of TGFBR1 or TGFBR2. It is characterized by the triad of 1) aortic aneurysms and arterial tortuosity, 2) orbital hypertelorism, and 3) craniofacial abnormalities. Up to 98% of LDS patients will have aneurysmal dilatation of the aortic root (Loeys et al. 2006). Because aortic dissection and rupture can result, surgical intervention is generally recommended and therefore a Bentall aortic root replacement was originally performed (Loeys et al. 2006). Pseudoaneurysm following Bentall procedure is a well-documented complication with reported incidence as high as 8–10% (Kouchoukos et al. 1991). Our patient was likely at higher baseline risk due to his underlying LDS. Generally, documented cases in the literature describe repeat surgical intervention to avoid further rupture (Schmoker and Miller 2002). Initially, a conservative approach was taken for our patient; however, serial echocardiography and gated cardiac CT demonstrated progressive enlargement of the pseudoaneurysm. The benefits and risks of endovascular management must be weighed carefully compared to open surgical repair with the patient’s specific clinical situation taken into account. There are several reports of minimally invasive methods for occlusion of aortic pseudoaneurysms with coils, vascular plug devices, as well as catheter directed thrombin injection (Komanapalli et al. 2005). These have not been proven safer compared to traditional open surgical repair, and complications such as aneurysm recurrence (Hibino et al. 2018), aorto-pulmonary fistula (Alameddine and Alimov 2016), as well as transient ischemic attack (in the case of thrombin injection (Lin et al. 2001)) have been reported. Direct percutaneous puncture as well as transapical approaches have also been described (Hadjivassiliou et al. 2017; Parhar et al. 2019); however this was felt to not be appropriate in our patient due to the position and orientation of the pseudoaneurysm. Transarterial coil embolization of aortic root pseudoaneurysms has not been previously described in patients with LDS. The main technical challenge was stable cannulation of the pseudoaneurysm, which took several attempts at selecting the appropriate guiding catheter. Once stable access was achieved, advancing the microcatheter system into the pseudoaneurysm for the duration of the coiling proceeded without difficulty. Its narrow neck proved ideal to achieving optimal packing and subsequent cure.

## Conclusion

We describe successful transarterial coil embolization of an aortic root anastomotic pseudoaneurysm in a patient with LDS post Bentall procedure. Transarterial coil embolization for aortic root pseudoaneurysms may provide an alternative option compared to open surgical repair for patients who are deemed high risk for repeat surgical intervention, such as those with connective tissue disease.

## Data Availability

The datasets used and/or analysed during the current study are available from the corresponding author on reasonable request.
